# Correction: Electrical Wave Propagation in an Anisotropic Model of the Left Ventricle Based on Analytical Description of Cardiac Architecture

**DOI:** 10.1371/journal.pone.0101611

**Published:** 2014-06-24

**Authors:** The PLOS ONE Staff

## Notice of Republication

This article was republished on June 5, 2014, to correct the images of Figures 6 and 7, which were switched during the production process. The publisher apologizes for the error. Please download this article again to view the correct version. The originally published, uncorrected article and the republished, corrected article are provided here for reference.

## Supporting Information

File S1 Originally published, uncorrected article.
(PDF)

File S2 Republished, corrected article.
(PDF)
