# Supplementary material for: Correction: Electrical Wave Propagation in an Anisotropic Model of the Left Ventricle Based on Analytical Description of Cardiac Architecture
Source: PLoS One. 2014 Jun 24;9(6):e101611. doi: 10.1371/journal.pone.0101611 (PMC4069158; doi:10.1371/journal.pone.0101611)
Supplement: File S1 — Originally published, uncorrected article. (PDF) [file pone.0101611.s001.pdf]

PLOS | ONE

Electrical Wave Propagation in an Anisotropic Model of the Left Ventricle Based on Analytical Description of Cardiac Architecture

Sergey F. Pravdin[1,2,3]*, Hans Dierckx[3], Leonid B. Katsnelson[2,4], Olga Solovyova[2,4], Vladimir S. Markhasin[2,4], Alexander V. Panfilov[3,5]*

1 Function Approximation Theory Department, Institute of Mathematics and Mechanics, Ekaterinburg, Russia, **2** Laboratory of Mathematical Physiology, Institute of Immunology and Physiology, Ekaterinburg, Russia, **3** Department of Physics and Astronomy, Faculty of Sciences, Ghent University, Ghent, Belgium, **4** Ural Federal University, Ekaterinburg, Russia, **5** Moscow Institute of Physics and Technology (State University), Dolgoprudny, Moscow Region, Russia

Abstract

We develop a numerical approach based on our recent analytical model of fiber structure in the left ventricle of the human heart. A special curvilinear coordinate system is proposed to analytically include realistic ventricular shape and myofiber directions. With this anatomical model, electrophysiological simulations can be performed on a rectangular coordinate grid. We apply our method to study the effect of fiber rotation and electrical anisotropy of cardiac tissue (i.e., the ratio of the conductivity coefficients along and across the myocardial fibers) on wave propagation using the ten Tusscher–Panfilov (2006) ionic model for human ventricular cells. We show that fiber rotation increases the speed of cardiac activation and attenuates the effects of anisotropy. Our results show that the fiber rotation in the heart is an important factor underlying cardiac excitation. We also study scroll wave dynamics in our model and show the drift of a scroll wave filament whose velocity depends non-monotonically on the fiber rotation angle; the period of scroll wave rotation decreases with an increase of the fiber rotation angle; an increase in anisotropy may cause the breakup of a scroll wave, similar to the mother rotor mechanism of ventricular fibrillation.

Citation: Pravdin SF, Dierckx H, Katsnelson LB, Solovyova O, Markhasin VS, et al. (2014) Electrical Wave Propagation in an Anisotropic Model of the Left Ventricle Based on Analytical Description of Cardiac Architecture. PLoS ONE 9(5): e93617. doi:10.1371/journal.pone.0093617

Editor: Alena Talkachova, University of Minnesota, United States of America

Received October 3, 2013; **Accepted** February 27, 2014; **Published** May 9, 2014

Copyright: © 2014 Pravdin et al. This is an open-access article distributed under the terms of the Creative Commons Attribution License, which permits unrestricted use, distribution, and reproduction in any medium, provided the original author and source are credited.

Funding: This work was supported by the Presidium of Urals Branch of the Russian Academy of Sciences (project 12-M-14-2009), the Flemish Community of Belgium (HD's fellowship and grant 1F2B8M/JDW/2010-2011/10-BTL-RUS-01), Ghent University (grant 01SF1511), the Agreement 211 between the Government of RF and UrFU #02.A03.21.0006, the program "State Support of the Leading Scientific Schools" (NS-4538.2014.1), the Russian Foundation for Basic Research (grant 13-01-96048) and the Government of the Sverdlovsk Region. The funders had no role in study design, data collection and analysis, decision to publish, or preparation of the manuscript.

Competing Interests: A.V. Panfilov is a PLOS ONE Editorial Board member. This does not alter the authors' adherence to all the PLOS ONE policies on sharing data and materials.

* E-mail: sergey.pravdin@ugent.be (SFP); alexander.panfilov@ugent.be (AVP)

Introduction

The modeling of cardiac electrical function is a well-established area of research that began with early models of cardiac cells developed by D. Noble [1].

The importance of modeling in cardiology comes from the widespread prevalence of cardiac disease. For example, sudden cardiac death is the leading cause of death in the industrialized world, accounting for more than 300,000 victims annually in the US alone [2]. In most cases, sudden cardiac death is a result of cardiac arrhythmias that occur in the ventricles of the human heart [2].

When studying cardiac arrhythmias, it is important to understand that they often occur at the level of the whole organ and in these situations cannot be reproduced in single cells. Therefore, it is very important to model cardiac arrhythmias at the tissue level, preferably using an anatomically accurate representation of the heart. Compared to modeling at the single-cell level, anatomical modeling started much more recently [3,4]. Using anatomical models, researchers have been able to obtain important results on the 3-D organization of cardiac arrhythmias in animal [5] and human [6] hearts. Moreover, the defibrillation process has been investigated [7], and the effects of mechano-electrical coupling on cardiac propagation have recently been modeled[8,9]. Multi-scale anatomical cardiac modeling is becoming increasingly prominent in medical and pharmaceutical research [10].

In a broad sense, an anatomical model of the heart is a combination of models of cardiac cells and anatomical data. The development of models of the electrical and mechanical functions of cardiac cells is a well-established area of research, and many models have been developed, including models of the human cardiac cells [11–20]. The anatomical data necessary for cardiac modeling include not only the heart's geometry but also its anisotropic properties. Although the geometry of the heart can be obtained from routine clinical procedures such as MRI or CT scans [21,22], anisotropy data are much more challenging to acquire. Currently, the acquisition can be done on explanted hearts only, using either direct histological measurements or time-demanding DT-MRI scans [23–26]. In addition to experimental noise, even perfect measurements will grant only the particular anisotropy of the imaged heart. Thus, to study the effects of

anisotropy on wave propagation, one needs to vary the anisotropic properties and to separate the anisotropy effects from other factors. All these questions can be addressed with the development of models that account for the anisotropy of the heart using analytical or numerical tools.

In a previous article [27], we described an axisymmetric model of the left ventricle (LV) of the human heart. In the model, we represented the LV shape (including positions of cardiac fibers) as analytical functions of special curvilinear coordinates defined on a rectangular domain. Our model allowed the generation of not only a default architecture of anisotropy closest to the reality but also intermediate architectures that can be used to study the effects of any specific element of anisotropy on wave propagation in the heart.

In this paper, we build on our previous approach in two ways. First, we develop a numeral scheme for the integration of equations for wave propagation in our anatomical model of the LV, which is the best possible way to account for anisotropy. In particular, we develop a model on a rectangular domain and represent anisotropy and the LV shape by means of parameter changes. Second, we vary the geometry and anisotropy parameters to study how the rotation of the fiber orientation affects wave propagation and show that rotational anisotropy accelerates the spread of electrical excitation in the heart. We also study the behavior of scroll waves and their filaments. We show that the scroll waves drift and we calculate their drift velocity and period of rotation depending on the fiber rotation angle and the diffusion coefficients ratio.

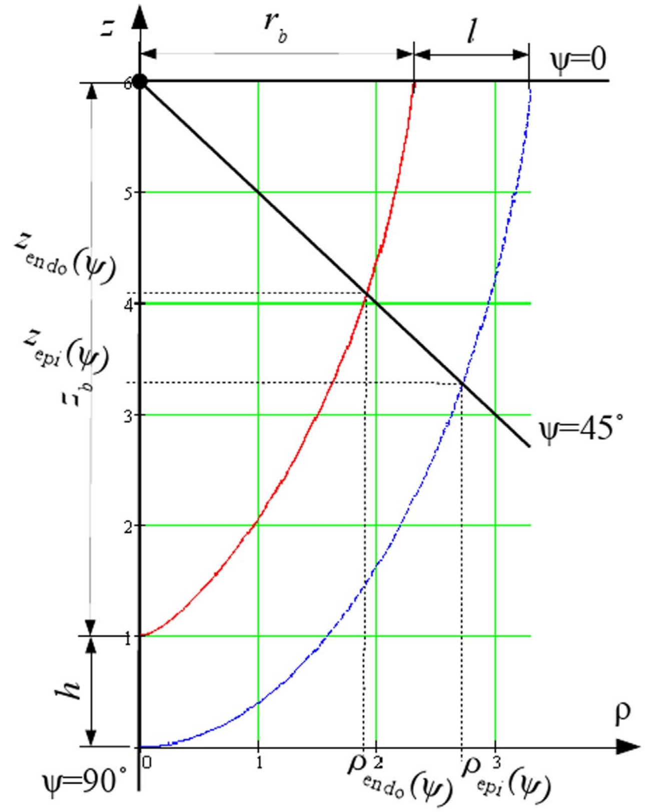

Figure 1. A radial section of the endocardial (solid red line) and epicardial (dashed blue line) surfaces of the LV model, from [27].
doi:10.1371/journal.pone.0093617.g001

Model Description

Geometrical model of the LV

In our model, the LV is represented as a body of revolution around the vertical axis Oz with the shape fitted to experimental data (for details, see [27]). A section of the LV is shown in Fig. 1. The rotation of the blue line delineates the epicardial surface, while the rotation of the red line yields the endocardial surface of the LV.

In our model, each point of the LV has three local coordinates (γ, ψ, ϕ). The coordinate γ ($\gamma_0 \leq \gamma \leq \gamma_1$) gives us points between the endo- and epicardial surfaces in Fig. 1, i.e., it is a measure for transmural depth; the coordinate ψ ($0 \leq \psi \leq \pi/2$) is explained in Fig. 1; and the coordinate ϕ ($0 \leq \phi < 2\pi$) is the rotation angle around the vertical axis Oz. The local coordinates are linked with the cylindrical coordinate system (CS) (ρ, φ, z) by the following formulae:

$$\rho = (r_b + (1-\gamma)l)\epsilon_c(\psi), \quad (1)$$

$$\varphi = \phi,$$

$$z = (z_b + (1-\gamma)h)(1 - \sin\psi) + (\gamma - 1)h, \quad (2)$$

with:

$$\epsilon_c = \epsilon \cos\psi + (1-\epsilon)(1 - \sin\psi).$$

Below, we also use

$$\epsilon_s = \epsilon \sin\psi + (1-\epsilon)\cos\psi.$$

The physical meaning of the parameters is as follows: r_b is the LV cavity radius on the LV equator, z_b is the LV cavity depth, l is the LV wall thickness on the LV equator, h is the LV wall thickness on the LV apex, and $\epsilon \in [0,1]$ is a dimensionless parameter influencing the conicity-ellipticity characteristic of the LV shape. In this system, $\gamma = \gamma_0$ gives the epicardium and $\gamma = \gamma_1$ gives the endocardium; the value $\psi = 0$ describes the upper (basal) boundary of the LV.

Following Pettigrew's idea [28] about spiral surfaces and semicircle chords mapping on the surfaces, our LV model consists of spiral surfaces on which a set of curves is defined. The details are described in our previous work [27] and are summarized in Appendices A (spiral surfaces construction) and B (fiber equations). In Figs. 2 and 3, we show common views of spiral surfaces and the fibers inside them.

We demonstrated in [27] that the model approximates the real fiber orientation field in the LV reasonably well. A comparison of true fiber angle α and helix angle α_1 with MRI data showed that fiber architecture in the equatorial region of the heart was well reproduced in our anatomical model. In the middle (by height) and apical areas, the angles were reproduced both qualitatively and quantitatively well; the difference between the model and the experimental data was not more than 25°.

Overall, we can consider the anatomical model as a map from a rectangular domain $\gamma_0 \leq \gamma \leq \gamma_1$; $0 \leq \psi \leq \pi/2$; $0 \leq \phi < 2\pi$ to the shape of LV with anisotropy explicitly given by Eqs. (B.1)–(B.3). The total fiber rotation angle $\Delta\alpha_1$ is defined as the difference between the epicardial and endocardial helix angles α_1 measured at the LV basal zone $\psi = \pi/8$ (see [29] for details). It can be varied by changing the values of the parameters γ_0 and γ_1:

$$\alpha_1(\gamma^*,\psi^*) = (p,\widehat{\mathrm{pr}_n}v)|_{\gamma=\gamma^*,\psi=\psi^*,\Phi=0},$$
$$\Delta\alpha_1(\gamma_0,\gamma_1) = \alpha_1(\gamma_1,\pi/8) - \alpha_1(\gamma_0,\pi/8), \quad (3)$$

where $\mathbf{p}$ is the tangent vector to the parallel $\gamma = \text{const}$, $\psi = \text{const}$ at the point $\gamma = \gamma^*$, $\psi = \psi^*$, $\phi = 0$ (the geometric model is axisymmetric; therefore the choice of ϕ is arbitrary); $\mathbf{n}$ is the normal vector to the epicardium passing through the same point; $\mathbf{v}$ is the fiber direction vector (it is defined by Eq. (B.1)–(B.3)); pr is the projection operator; and $(\widehat{\mathbf{u},\mathbf{w}})$ denotes the angle between the vectors $\mathbf{u}$ and $\mathbf{w}$. We have chosen the value $\psi = \pi/8$ because in this case, the total fiber rotation angle changes uniformly enough depending on the values of $\gamma_{0,1}$ we use (see Table 1 and section "Parameter values" below). For short, below we will denote $\Delta\alpha_1(\gamma_0, \gamma_1)$ as α without argument. We use it in the present study to investigate the effect of fiber rotation on wave propagation in the heart.

Electrophysiological model

To describe the excitation of cardiac tissue, we use the detailed ionic model for human ventricular cells from [6,11]. The model uses reaction-diffusion equations to describe the evolution of the transmembrane potential $u = u(\mathbf{r}, t)$:

$$\frac{\partial u}{\partial t} = \mathrm{div}(\mathbf{D}\ \mathrm{grad}\ u) - \frac{I_{ion}}{C_m}, \quad (4)$$

$$I_{ion} = I_{Kr} + I_{Ks} + I_{K1} + I_{to} + I_{Na} + I_{bNa} + I_{CaL} +$$
$$I_{bCa} + I_{NaK} + I_{NaCa} + I_{pCa} + I_{pK}. \quad (5)$$

Here, the intracellular processes are captured by $I_{ion} = I_{ion}(\mathbf{r}, t)$ which is the sum of the ionic transmembrane currents; C_m is the capacitance of the cell membrane. The locally varying diffusion matrix $\mathbf{D}$ accounts for myofiber anisotropy. As in [6], the diffusion matrix $\mathbf{D} = (D^{ij})$ was computed using the following formula

$$D^{ij} = D_2\delta_{i,j} + (D_1 - D_2)v_i v_j, \quad (6)$$

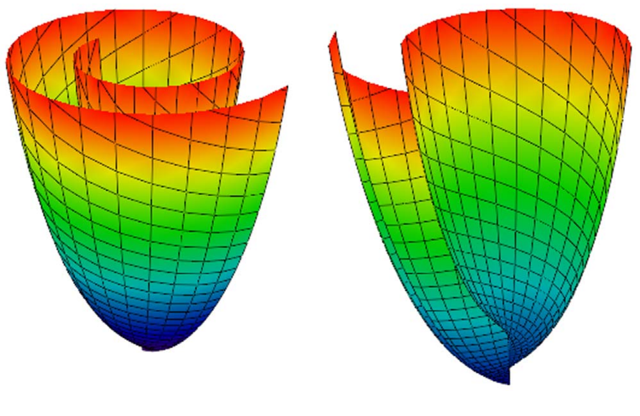

Figure 2. A spiral surface. The lines on the surface have equations $\rho = \text{const}$ and $\phi = \text{const}$. Color corresponds to height (z coordinate).
doi:10.1371/journal.pone.0093617.g002

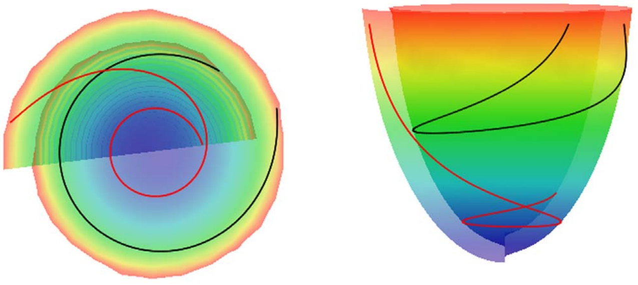

Figure 3. A spiral surface viewed from the top (left panel) and side (right panel). Two myofibers are displayed in red and black.
doi:10.1371/journal.pone.0093617.g003

where D_1 and D_2 are the diffusion coefficients along and across the fibers, $\mathbf{v}$ is the unit vector of fiber direction, i, j are Cartesian indices, and $\delta_{i,j}$ is the Kronecker symbol.

Numerical integration scheme and boundary conditions

The aim in this paper is to present a numerical procedure that allows us to use the analytical representation of cardiac anatomy and anisotropy described in the previous section. In particular, as our anatomical model is just a map from a rectangular domain $\gamma_0 \le \gamma \le \gamma_1$; $0 \le \psi \le \pi/2$; $0 \le \phi < 2\pi$ to the shape of LV, we can formulate our approach in that rectangular domain. The shape of the heart, as well as anisotropy, will then be a curvilinear coordinate system (1)–(2) defined on that domain. We need to recalculate Eq. (4) with no-flux boundary conditions in those coordinates. A long computation presented in appendix C results in the following expression of the diffusion term:

$$\mathrm{div}(\mathbf{D}\ \mathrm{grad}\ u) = \sum_k p_k \cdot \frac{\partial u}{\partial \xi_k} + \sum_{k,l} q_{kl} \cdot \frac{\partial^2 u}{\partial \xi_k\, \partial \xi_l}, \quad (7)$$

where $\xi_1 = \gamma$, $\xi_2 = \psi$, $\xi_3 = \phi$, and p_k and q_{kl} are coefficients given by the explicit analytical Eqs. (C.12) and (C.13) that depend only on the geometry of the LV and on the diffusion matrix. This representation is similar to that presented by Sridhar et al. in [30]. However, in our work, it was done for the 3-D case and for a special form of the diffusion matrix (see Eq. (6)).

For numerical integration of the model (B.1)–(B.3), (4), (5), we use the explicit finite difference method on a discrete grid in the (γ, ψ, ϕ) space. We initially use a uniform grid with the γ-indices of the nodes denoted as $i = 0, 1, \ldots n_\gamma$; the ψ-indices as $j = 0, 1, \ldots n_\psi$; and the ϕ-indices as $k = 0, 1, \ldots n_\phi$.

Although this grid is uniform in the (γ, ψ, ϕ) space, it is non-uniform in real space because distances between the grid points substantially decrease when ψ approaches $\pi/2$, which is similar to the situation at the pole in a polar CS. Note, however, that even the cubic Cartesian lattice in real space is not uniform with respect to anisotropic diffusion due to the curved space interpretation of anisotropy [31,32]. To account for this problem, we exclude some points from our uniform grid in the following way. We first choose a threshold value of distance $d_{\min}$. Then, at $\gamma = \gamma_1$ (i.e., at the epicardial surface) and any given $\psi = \psi_j$, we calculate the distances between the node at $\phi = 0$: ($\gamma = \gamma_1$, $\psi = \psi_j$, $\phi = 0$) and node ($\gamma = \gamma_1$, $\psi = \psi_j$, $\phi = \phi_k$). We find minimal k satisfying two conditions: (1) the distance to the k-node from the node at $\phi = 0$ is more than the threshold value $d_{\min}$; and (2) k is a divisor of n_ϕ. We denote this number as K_j (as it depends on ψ_j). If ψ is far from $\pi/2$, then $K_j = 1$ and we use all nodes of our uniform (γ_i, ψ_j, ϕ_k) grid. When ψ approaches the value of $\pi/2$, $K_j > 1$ and we drop all nodes between

Table 1. Dependence of the total fiber angle on the model parameters $\gamma_{0,1}$.

Model	γ_0	γ_1	The helix angle near the base at the epicardium	the endocardium	The total fiber rotation angle α
1	0.3	0.55	$-13°$	$3°$	$16°$
2	0.2	0.7	$-40°$	$29°$	$69°$
3	0.1	0.85	$-69°$	$64°$	$133°$
4	0	1	$-87°$	$87°$	$174°$

doi:10.1371/journal.pone.0093617.t001

$(\gamma=\gamma_1, \psi=\psi_j, \phi=0)$ and $(\gamma=\gamma_1, \psi=\psi_j, \phi=K_j)$, then the next node will be $(\gamma=\gamma_1, \psi=\psi_j, \phi=2K_j)$ etc. Thus, only nodes with the ϕ-indices $0, K_j, 2K_j, \ldots$ will be taken for evaluation of the Laplacian. After each time step, we compute values for all variables of our model in the omitted nodes using linear interpolation. With this approach, we reduce the number of grid elements in the ϕ-direction in the apical region; otherwise, we would have needed to lower the maximal time step in our explicit integration scheme, which would have slowed our computations significantly.

The no-flux boundary condition in our problem is

$$\mathbf{nD}\ \mathrm{grad}\ u=0, \tag{8}$$

where $\mathbf{n}$ is a normal to surface. We rewrite this equation in our special CS and obtain the following expression:

$$c_\gamma \frac{\partial u}{\partial \gamma}+c_\psi \frac{\partial u}{\partial \psi}+c_\Phi \frac{\partial u}{\partial \Phi}=0, \tag{9}$$

where c_γ, c_ψ, c_ϕ are coefficients given by Eqs. (D.14) and (D.16) in Appendix D. In order to satisfy the boundary condition, we add nodes at the domain boundaries in the following way. In the special CS, the domain of integration is a rectangle (with periodic boundary in ϕ), and at $\psi=\pi/2$ we have a pole (i.e., also no boundary). Therefore, we have only three boundaries, namely at $\gamma=\gamma_0$ (i.e., $i=0$), $\gamma=\gamma_1$ $(i=n_\gamma)$, and $\psi=0$ $(j=0)$. Fictitious layers with $(-1, j, k)$, $(n_\gamma+1, j, k)$, and $(i, -1, k)$ are added. We then solve equation (9) on the three boundary surfaces to find values in the added nodes. Subsequently, we can compute Laplacian at all other nodes in the domain using, if necessary, the values in the additional nodes. Due to this procedure, all the nodes lying on the boundaries will satisfy the boundary conditions.

We have programmed this approach using C in the CodeBlocks IDE, a Mingw compiler. The calculations were performed in operating systems Windows 7 and Linux. The OpenMP and MPI technologies have been used for parallelization, and Paraview and Irfan have been used for visualisation. The formal parameters of our numerical scheme have been given in the methods section above. Such an approach allows us to compute various regimes of wave propagation in a model of LV with good representation of boundary conditions and to study various effects of anisotropy on wave propagation patterns.

We also studied the dynamics of scroll waves using the ten Tusscher–Noble–Noble–Panfilov (TNNP) model [11] and various anisotropy parameters. We initiated a scroll wave using the S1–S2 stimulation protocol and studied its dynamics 12 s. For drift velocity and rotation period calculations, we take into account only the last 8 seconds of the simulations to exclude transient processes. We calculated average periods in the section $\phi=0$, that is, $x=\rho>0, y=0$. Filaments were analyzed as reported in [6,33]. Finally, we computed their average velocity v in the Cartesian CS.

Parameter values

We used the following parameters from Table 2 in [29]: the LV equatorial radius $r_b^e=23$ mm, the equatorial wall thickness $\ell^e=10$ mm, the LV cavity depth $z_b^e=53$ mm, the apical wall thickness $h^e=7$ mm, the conicity-ellipticity parameter $\epsilon=0.9$, and the spiral surface torsion angle $\Phi^e_{\max}=3\pi$ (see Fig. 3c in [29]). The threshold distance between the adjacent nodes $d_{\min}$ was set to 0.3 mm. Currently, the first four parameters are measured using modern experimental techniques such as MRI (see [34–36]). The values used in our paper are in agreement with these experimental data.

Our mesh had a distance of 0.2 to 0.3 mm between the nodes and before the deletion of the nodes described above had $n_\gamma=40$, $n_\psi=300$, $n_\psi=800$. The diffusion coefficient along the fibers was $D_1=0.3$ mm^2/ms. The diffusion coefficient across the fibers D_2 was varied between different experiments depending on whether we modeled isotropy or anisotropy.

We applied our approach to study the effect of anisotropy on the spread of excitation in the heart. In particular, we initiated a wave at several locations and studied how the wave arrival time depends on the two main features of anisotropy. Our first anisotropy parameter was the ratio of the diffusion coefficients D_1:D_2 along and across the fibers. Also, we independently varied the total rotation angle of fibers through the myocardial wall by adjusting γ_0, γ_1 and keeping wall thickness constant. We also compared our results with the spread of excitation in an isotropic model of the LV where $D_1=D_2$.

The dependency of wave velocity on the direction of propagation in the heart was measured in [37–39]. Experimental data show that the ratio of longitudinal to transverse conduction velocities ranges between 3 [38] and 2.1 [39]. Since $D\propto c^2$, where c is wave velocity (see, e.g., [40]), we used the ratios D_1:$D_2=1$:0.111 and D_1:$D_2=1$:0.25, which correspond to the experimental data. These anisotropy ratios were also used in the modeling studies [33,41–44].

For point stimulation, we increased the value of the variable u from the resting potential of -86.2 mV to $u=0$ mV at the first time step in small regions located at three different sites. In the A series, it was a small epicardial region at the apex; in the B series, at the centre of LV epicardium; in the C series, at the centre LV endocardium (see Table 2).

In this paper, we study the effect of the fiber rotation on the spread of excitation. With this purpose, we generated a series of LV models that differ in the total fiber rotation angle through the myocardial wall. The parameters of the model are listed in Table 1. Note that although the values of γ_0 and γ_1 differ between the models, they affect only fiber rotation, and the LV geometry is

Table 2. The initial excitation areas.

Series	γ-indices	ψ-indices	ϕ-indices	area
A	$i \geq n_\gamma - 4$	$j \geq n_\psi - 4$	all	apical epicardium
B	$i \geq n_\gamma - 4$	$\lvert j-(n_\psi/2)\rvert \leq 2$	$k \leq 4$	central epicardium
C	$i \leq 4$	$\lvert j-(n_\psi/2)\rvert \leq 2$	$k \leq 4$	central endocardium

doi:10.1371/journal.pone.0093617.t002

exactly the same for all models due to the rescaling procedure described in Sec. 1.4 in our previous article [27]. Also note that the change in fiber rotation results in the change in fiber angle at the epicardial surface (see column "α" of Table 1).

The time step was equal to 0.005 ms for the isotropic cases and 0.01 ms for the anisotropic ones.

We considered that a wave came to a node when the potential in the node was more than -80 mV the first time.

Numerical Results

Activation maps

Figure 4 shows the wave arrival time after stimulation of the small apical epicardial zone A for ratios D_1:D_2 equal to 1:0.111 or 1:0.25 (shown at the top of the figure) and four different rotation angles of the fibers, which are displayed in the left column. We see that in this first example, all the figures are axisymmetrical, which is a consequence of the axisymmetric properties of our model and the initial conditions.

We observe that for the low rotation angle (the upper row), the speed of the upward wave propagation for the diffusion coefficient ratio of 1:0.111 is substantially smaller than that for the ratio of 1:0.25. However, if the fiber rotation angle increases (the lower rows), the difference in the speed between the two anisotropy ratios decreases. For the rotation angle of 174° (the lower row), the excitation patterns for both anisotropy ratios become close to each other. Thus, we observe that fiber rotation increases the velocity of the spread of excitation and also decreases the effect of anisotropy.

Let us now consider the case of lateral stimulation for a given ratio of the diffusion coefficients of 1:0.111; the results for epi- and endocardial stimulation are presented in Figs. 5 and 6. After epicardial stimulation, the wave initially follows the fiber direction. In Fig. 5, we note a displacement of the early activation zones (red) due to the change in fiber direction at the epicardium in our anatomical model (see column 4 of Table 1). However, for endocardial stimulation (Fig. 6, the first column), the shift of the early activation zone (red) on the epicardium is attenuated by fiber rotation. As in Fig. 5, we see that an increase in the rotation angle causes a decrease in the arrival time. In addition, in the second column in Fig. 5, the excitation patterns have a clear V shape on the surface opposite the stimulation site, which is a mere consequence of the shape of the heart (see [3,4]).

Figs. 7 and 8 show the arrival time after lateral stimulation in a case of decreased anisotropy, i.e., for a diffusion coefficient ratio of 1:0.25. The excitation patterns resemble those from Fig. 5 and Fig. 6 respectively, with similar effects of fiber rotation on the epicardial stimulation and V-shaped patterns. Here, we also observe that an increase in the rotation angle decreases the overall excitation time. A compensating effect of fiber rotation on the degree of anisotropy can be noted. The difference between the corresponding panels in Fig. 5 and Fig. 7 (and also Fig. 6 and Fig. 8) is more pronounced for a lower fiber rotation rate (rotation angle 16°).

Average speed of excitation

Now let us quantify the effects of rotation and anisotropy on wave propagation. In order to do this, we use the following procedure. We group all points of the heart to bins differing by their "distance" from the stimulation point. We define the distance as the arrival time from the stimulation to a given point. To calculate the distances, we perform simulations in which we initiate a wave at the same locations as in Figs. 4–8. However, for the isotropic medium, we use a diffusion coefficient of $D_1 = D_2 = 0.3$ mm^2/ms. We generate 40 groups in which points differ in the arrival time by 2 ms. Then we determine the average arrival time for each of these groups for various anisotropic conditions and compare these arrival times to the arrival time in the isotropic model. As in the isotropic model, the velocity of wave propagation in all directions is the same; this dependence gives us the dependence of the wave arrival time on the distance from the stimulation point.

In Fig. 9, the red lines correspond to $\alpha = 174°$ and the black lines correspond to $\alpha = 16°$ fiber rotation angle in the LV wall. The

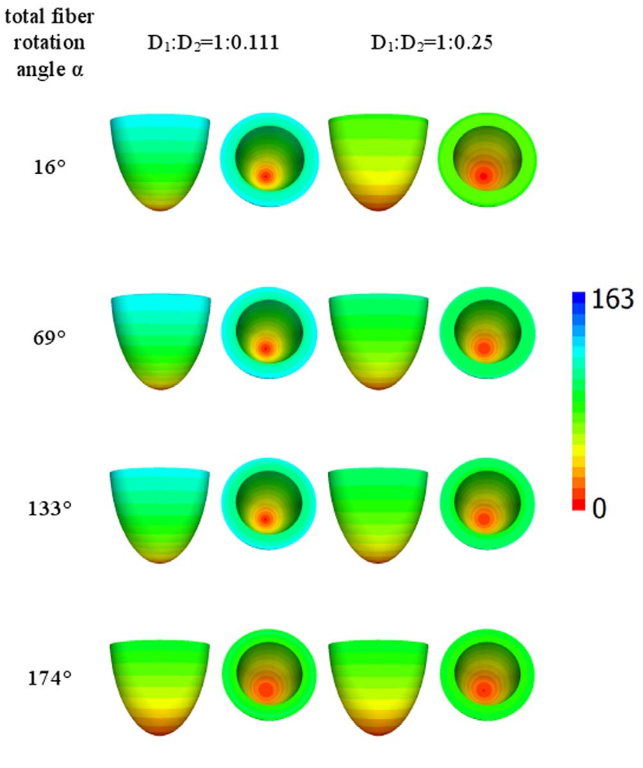

Figure 4. Arrival times, in ms, of the waves after point stimulation at the apex for various values of anisotropy and fiber rotation. The values of anisotropy are shown at the top of the figure and the values of the fiber rotation are shown in the left column. For details, see Table 1. Arrival times are color-coded in ms.
doi:10.1371/journal.pone.0093617.g004

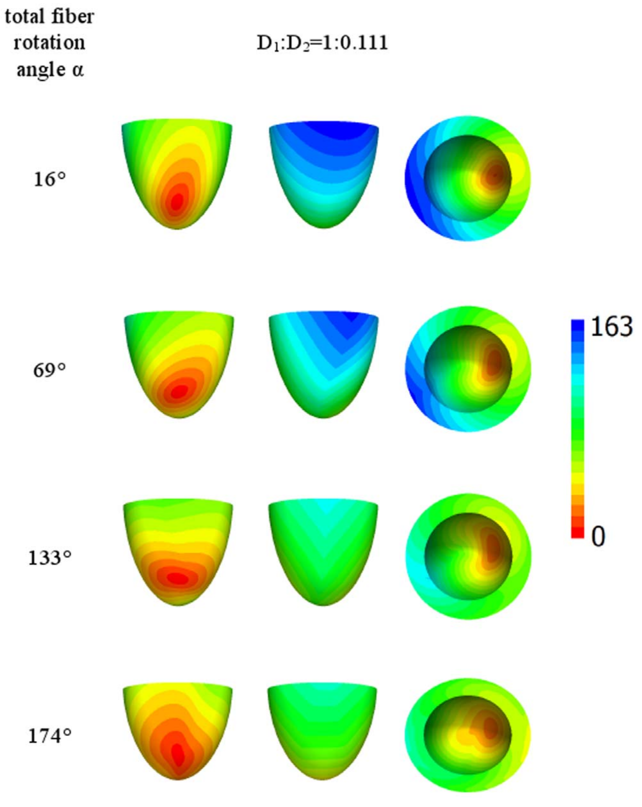

Figure 5. Arrival times, in ms, of the waves after point stimulation at the epicardial surface for a large anisotropy ratio D_1:D_2 = 1:0.111. The notation is the same as in Fig. 4.
doi:10.1371/journal.pone.0093617.g005

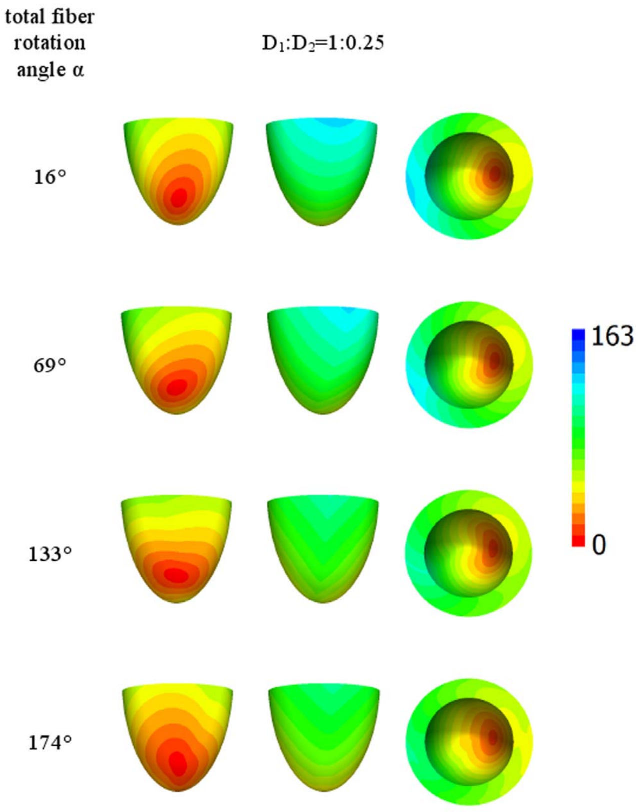

Figure 6. Arrival times, in ms, of the waves after point stimulation at the endocardial surface for a large anisotropy ratio D_1:D_2 = 1:0.111. The notation is the same as in Fig. 4.
doi:10.1371/journal.pone.0093617.g006

fact that the red lines are always located below the black lines shows that the increase of the fiber rotation angle results in faster wave propagation.

Also, all solid lines correspond to D_1:D_2 = 1:0.111, while dashed lines correspond to D_1:D_2 = 1:0.25. If we now compare the solid and dashed lines of the same color (third column in Fig. 9), we see that the red lines are closer to each other than the black lines. This indicates that in presence of higher fiber rotation (the red lines) the decrease of D_2 (i.e., solid vs. dashed) has a smaller effect on the arrival time. This once again illustrates that anisotropy is compensated for by the rotation of the fibers.

In addition, we see in Fig. 9 that the red lines always have a less steep slope than the corresponding black lines. As going from black to red shows an increase in fiber rotation, we can conclude that the increase in rotation makes propagation faster in all cases.

Scroll wave dynamics

We have also studied scroll wave dynamics for the same values of anisotropy and fiber rotation. We generated a single scroll wave located approximately at the middle between the apex and the base of the ventricle and studied its behavior for different model parameters γ_0, γ_1 and D_1:D_2. We found that anisotropy substantially affects the dynamics of scroll waves. In all cases, the increase in the fiber rotation angle results in a decrease in the period of scroll wave rotation (Fig. 10). We see that for D_1:D_2 = 1:0.25, when the fiber rotation angle is increased from 16° to 174°, the period drops significantly, from 277 to 257 ms. For D_1:D_2 = 1:0.111, we see similar dependency. In addition, the period for the same rotation angle for D_1:D_2 = 1:0.111 was slightly longer than for D_1:D_2 = 1:0.25.

The scroll wave dynamics was also substantially affected by the anisotropy. In all cases, we observed a drift of the filament (Fig. 11). The drift always had two components, both in the vertical (ψ) and circumferential (ϕ) directions. The total velocity of drift (Fig. 12A) was very small, about 1 mm/s, which is about 0.2 mm per rotation; however, the drift was monotonic and persistent. The value of velocity had no clear relationship with the rotation angle; for D_1:D_2 = 1:0.25, we see some tendency for velocity decrease with an increase in fiber rotation, while for D_1:D_2 = 1:0.111, the dependency is strongly non-monotonic and it is maximal for the intermediate values of the fiber rotation angle. The drift direction can be seen from the sign of the vertical and horizontal components of the velocity (Figs. 12B, C). Here again, the direction is affected by the rotation angle; however, we also did not find any clear tendency for either drift to the apex or base of the heart depending on the rotation angle.

For D_1:D_2 = 1:0.25, the initial scroll wave was always stable and did not break down to multiple scroll waves. For D_1:D_2 = 1:0.111, we did observe formation of the additional sources of excitation. However, in most cases, they appeared simultaneously at a substantial distance from the initial filament and not as a result of filament buckling and breakup due to rotational anisotropy in the way it was reported in [33]. The onset of new sources had a clear correlation with the fiber rotation angle (Fig. 13). We did not observe any instabilities for small and big α (models 1 and 4 in Table 1, Figs. 13A, D), however, for intermediate and large values of α (Figs. 13B, C), we observed new sources, and their number increased with the increase of the fiber rotation angle (compare panels B and C). Note that cases presented in panels B and C

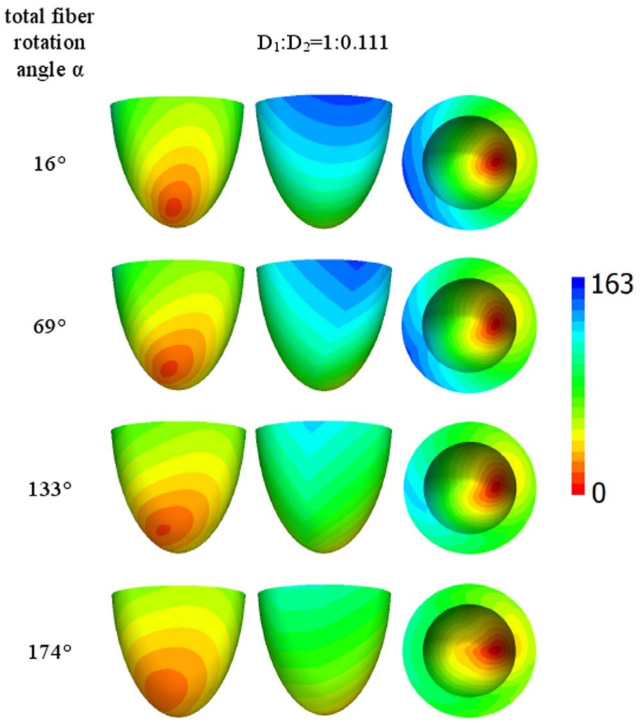

Figure 7. Arrival times, in ms, of the waves after point stimulation at the epicardial surface for an intermediate anisotropy ratio D_1:D_2 = 1:0.25. The notation is the same as in Fig. 4.
doi:10.1371/journal.pone.0093617.g007

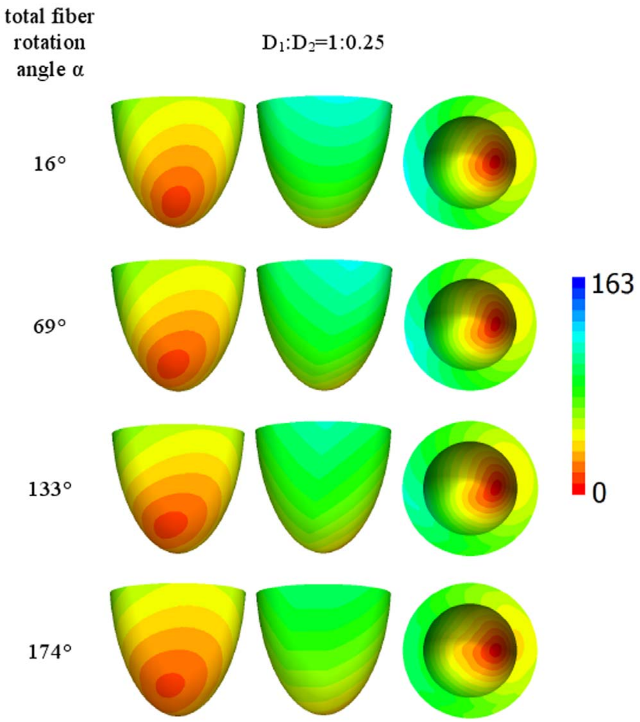

Figure 8. Arrival times, in ms, of the waves after point stimulation at the endocardial surface for an intermediate anisotropy ratio D_1:D_2 = 1:0.25. The notation is the same as in Fig. 4.
doi:10.1371/journal.pone.0093617.g008

correspond to the largest drift velocities of a scroll wave; thus, the onset of secondary filaments may be related to the filament drift.

We have also studied change in filament shape over time. For small values of total fiber angle α, the filament remained transmural, nearly straight, and stable. This case is shown in Fig. 13A. For intermediate α equal to 69° and 133°, the filament not only drifted faster but also deformed to a transmural S or L-shape (Fig. 13B). For larger values of α, the filament again had a nearly straight shape (Fig. 13D).

Discussion

In this paper, we used our recent anatomical model of the LV of the human heart using a special CS (γ, ψ, ϕ) which gives an explicit analytical map from a rectangular domain to the heart shape and fiber orientation field. This allowed us to represent the heart's geometry on a rectangular grid and explicitly write expressions for boundary conditions. This approach may be helpful for studies of any phenomena in which boundary effects are of great importance.

One important feature of our model is the possibility to change the properties of anisotropy. The most significant characteristic of LV anisotropy is the rotation of myocardial fibers through the myocardial wall. As shown in [45], relatively simple rule-based global models of LV myofiber directions yield no worse results than complicated image-based locally optimized models. Since any locally optimized model needs to be regular enough and the regularity requires smoothing and interpolation, no image-based model can be an untouched copy of a real heart.

We can change the degree of the fiber rotation in a consistent way and study its effect on normal and abnormal wave propagation in the heart. In this paper, we investigated two main features of wave propagation using the detailed human ventricular electrophysiological model: the distribution of effective excitation speed and the dynamics of transmural scroll waves.

In our study of the effect of the fiber rotation and anisotropy on wave propagation, the initial stimulation area was located at the apex and on the lateral epi- and endocardium of the LV. We found that the rotation of myocardial fibers accelerates the spread of excitation waves in the heart, which was explicitly demonstrated using models with different fiber rotation angles. This acceleration of wave propagation was discussed in [32]; it occurs because the wave can propagate with maximal speed in more directions with a larger rotation angle, which results in an overall faster wave propagation. Note that if the rotation angle is 2π or more, propagation with maximal speed will be possible in any direction, and in the limit of a large medium, the arrival time will be the same as in an isotropic medium with the velocity determined by the velocity along the fiber [32]. We were able to demonstrate this in an anatomical setup in which we explicitly changed the rotation of the fibers, while in [32] such an assessment was made for a single anisotropy configuration. While Young and Panfilov represented the tissue as a simple rectangular 3-D slab with plane–parallel fibers [32], we adopt a fully 3-D fiber architecture together with an LV shape. In this paper, we particularly considered a more realistic ventricular architecture and morphology that includes the following features:

1. A more realistic method for the fiber rotation angle around the axes [27], so called "Japanese-fan arrangement" [29]; and
2. A realistic change of the fiber rotation angle values due to the displacement of the transmural axis from the LV apex to the base [27].

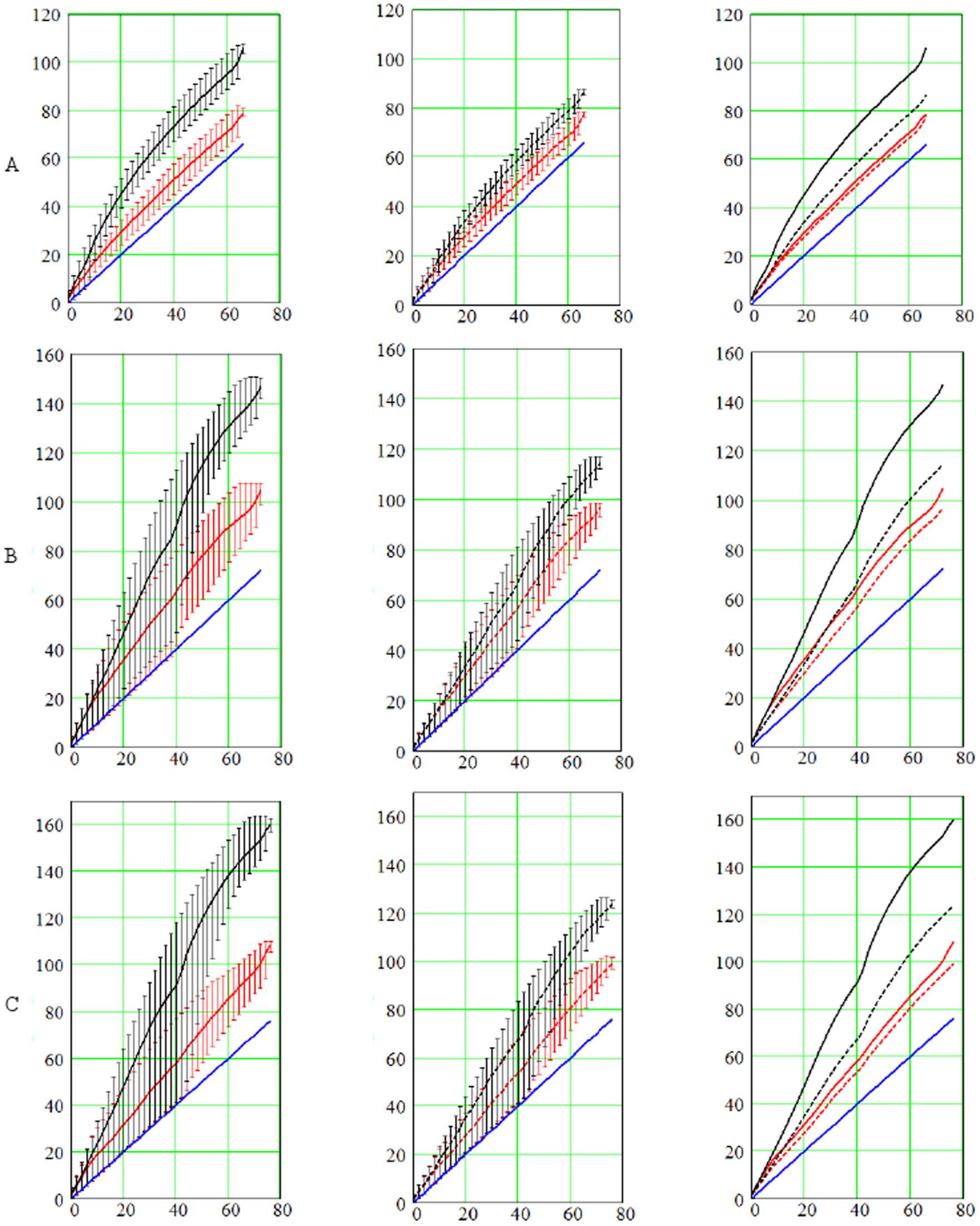

Figure 9. Arrival times, in ms, as a function of the distance from the stimulation point for the apical (*A*), epicardial (*B*), and endocardial (*C*) stimulation. The distance on the horizontal axis is measured in ms as the arrival time of the wave in the isotropic model (see text for more details). The red lines represent numerical experiments for total rotation angle $\alpha = 174^{\circ}$; the black lines represent for $\alpha = 16^{\circ}$; and the blue lines represent isotropy. The solid lines correspond to the case D_1:D_2 = 1:0.111, while the dashed lines correspond to the case D_1:D_2 = 1:0.25. The vertical segments display minimal and maximal arrival times in each group of nodes. The average, min, and max arrival times are displayed on the leftmost panels for D_1:D_2 = 1:0.111 and in the middle column for D_1:D_2 = 1:0.25. The right column compares the average arrival times.
doi:10.1371/journal.pone.0093617.g009

As in [32], our model also shows the faster excitation propagation with an increase in the fiber rotation angle and a decrease in the anisotropy.

A second set of results in this paper concerns the dynamics of transmural scroll waves. The negative correlation found for the rotation period versus fiber rotation angle in Fig. 10 is different from the observations in [46] made by Qu et al., who observed an increasing period with a faster fiber rotation rate. Note, however, that their simulations used in-plane fiber rotation, while we work with a 3-D ventricular geometry. Both complementary cases can

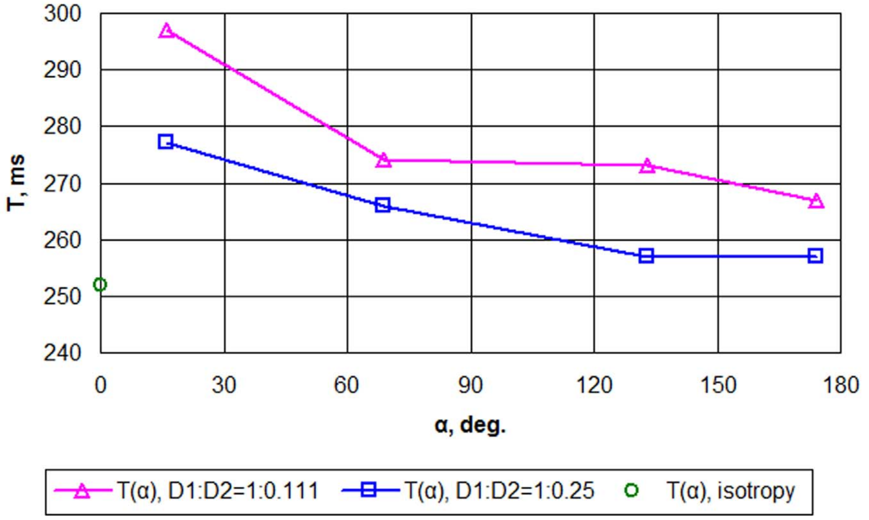

Figure 10. Scroll wave rotation period *T*, ms, as a function of total fiber rotation angle α, deg.
doi:10.1371/journal.pone.0093617.g010

be qualitatively explained on geometrical grounds. In [46], the period was only affected by fiber rotation, which causes negative intrinsic curvature $\mathcal{R}$ of the associated curved space [32]. By its definition, negative geometrical curvature represents a saddle-like space with positive angular deficit. More specifically, in a space with curvature $\mathcal{R}$, the circumference C of a circle (ball) with small radius r amounts to [47]

$$C \approx 2\pi r\left(1-\frac{\mathcal{R}r^2}{12}\right). \tag{10}$$

As the spiral tip in each cross-section needs to travel along a larger closed path of length C before completing a period, negative $\mathcal{R}$ is expected to increase the rotation period. Therefore, the trend found in [46] can be expected if the fiber rotation is the main determinant of the rotation period. In our present model, however, a transmural filament consists of spiral waves' tips in different layers of constant depth γ. These γ-surfaces are sphere-like and therefore have positive $\mathcal{R}$. Thus, under normal excitability, the sphericity of the LV will decrease the rotation period [48,49]. We conclude that in general, the rotation period of scroll waves in the heart may decrease or increase with increasing fiber rotation angle α, depending on the relative strengths of the competing effects of the fiber rotation rate and the extrinsic curvature (sphericity) of the LV cavity.

Regarding the non-monotonic dependence of filament drift velocity versus total rotation angle, we first note that the creation of secondary filaments in the regime of intermediate α is consistent with the clinical or experimental picture of a "mother rotor" during cardiac arrhythmias [50,51]. In such a scenario, the primary filament (mother rotor) remains stable and creates secondary sources that further disturb the electrical excitation of the heart, leading to cardiac arrest. In our simulations, we also see a similar situation with a stable mother rotor, which was always sustained until the end of the simulation time, and secondary excitation sources induced at some distance from it.

In addition to the direct simulation results detailed and discussed above, our anatomical model [27] coupled to detailed electrophysiological model [11] may prove useful in future studies for the following reasons. First, our model can be used to investigate the possible contribution of the LV geometry to the propagation of the excitation waves. In particular, in certain heart diseases (e.g. dilated and hypertrophic cardiomyopathy, eccentric

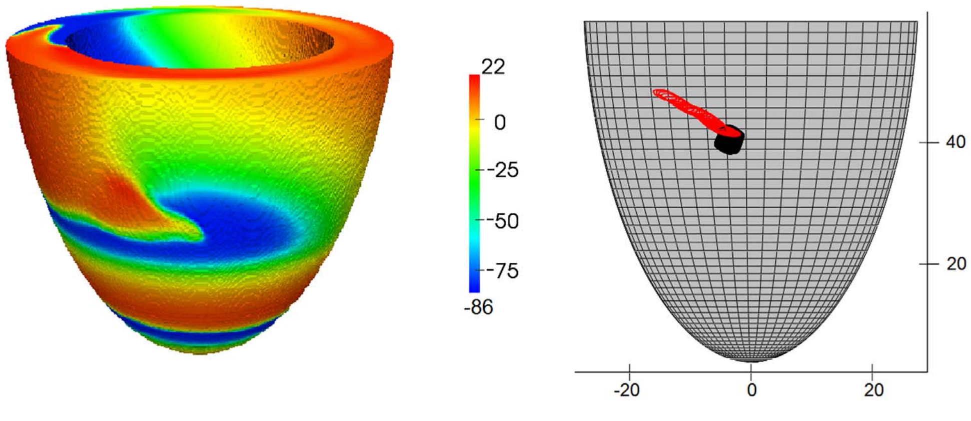

Figure 11. Potential, mV, on the LV surface during scroll wave rotation (left) and tip trajectory for D_1:D_2 = 1:0.111 (red line) and for D_1:D_2 = 1:0.25 (black line) (right). The results are shown for model 2 ($\gamma_0 = 0.2$, $\gamma_1 = 0.7$, see text and Table 1 for details).
doi:10.1371/journal.pone.0093617.g011

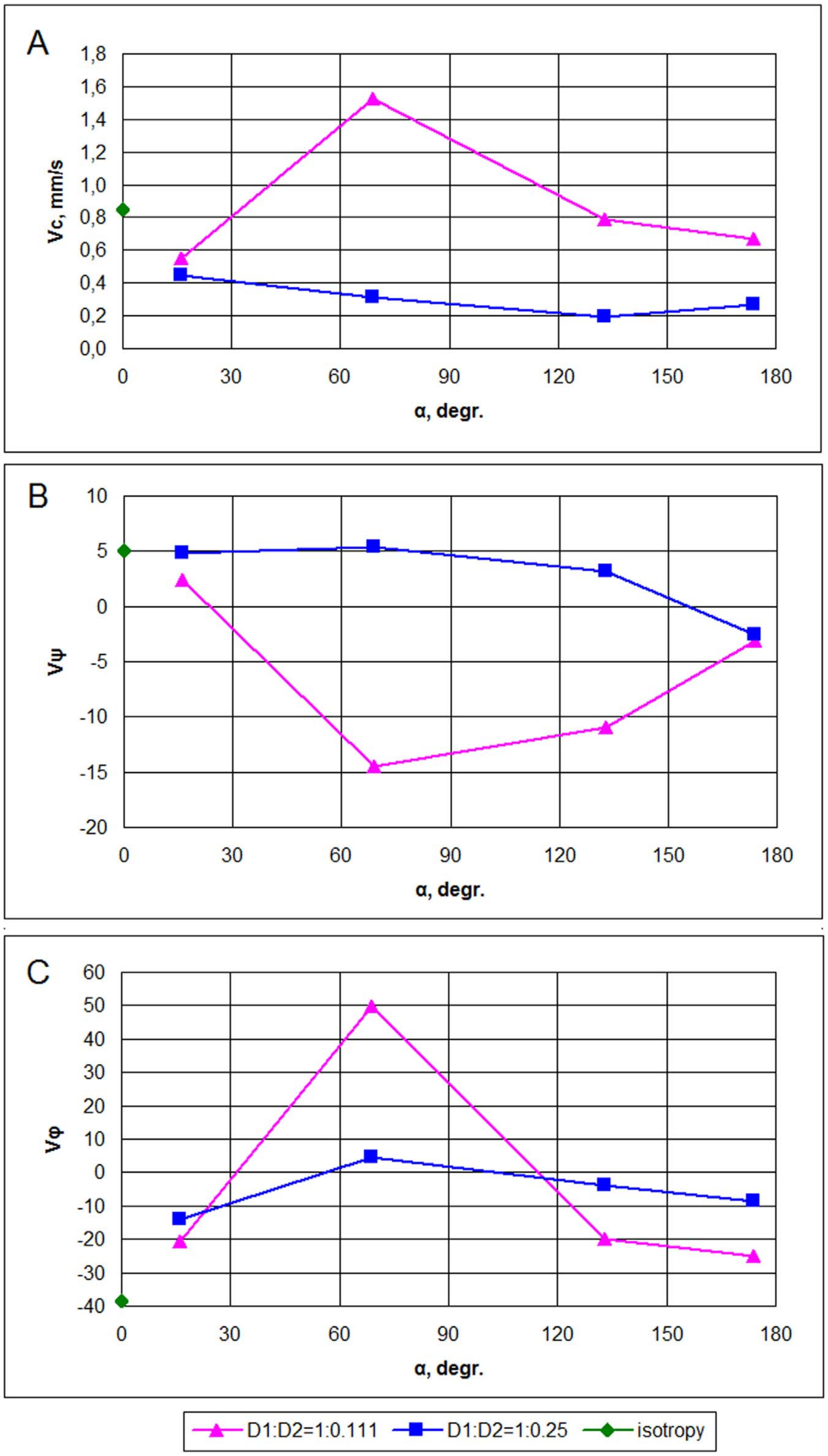

Figure 12. Velocity of scroll wave filament drift for the simulation of 8 s. Average filament velocity V_c, mm/s (A). Velocity components multiplied by 1000, per second: latitudinal component v_ψ (B) and longitudinal component v_ϕ (C).
doi:10.1371/journal.pone.0093617.g012

and concentric cardiac hypertrophy, etc., see chapter 8 in [52]), the shape of the ventricle becomes more spherical and the thickness of the wall also increases. Such changes in geometry can easily be accommodated in our model. In general, the study of the effects of the LV geometry on excitation seems to be of great importance because many cardiac pathologies tightly correlate with changes in the LV geometrical characteristics. The LV becomes more dilated near the apex and thicker near the base during stress-induced ("Takotsubo") cardiomyopathy, or transient apical ballooning syndrome (see chapter 8 in [52]). Such

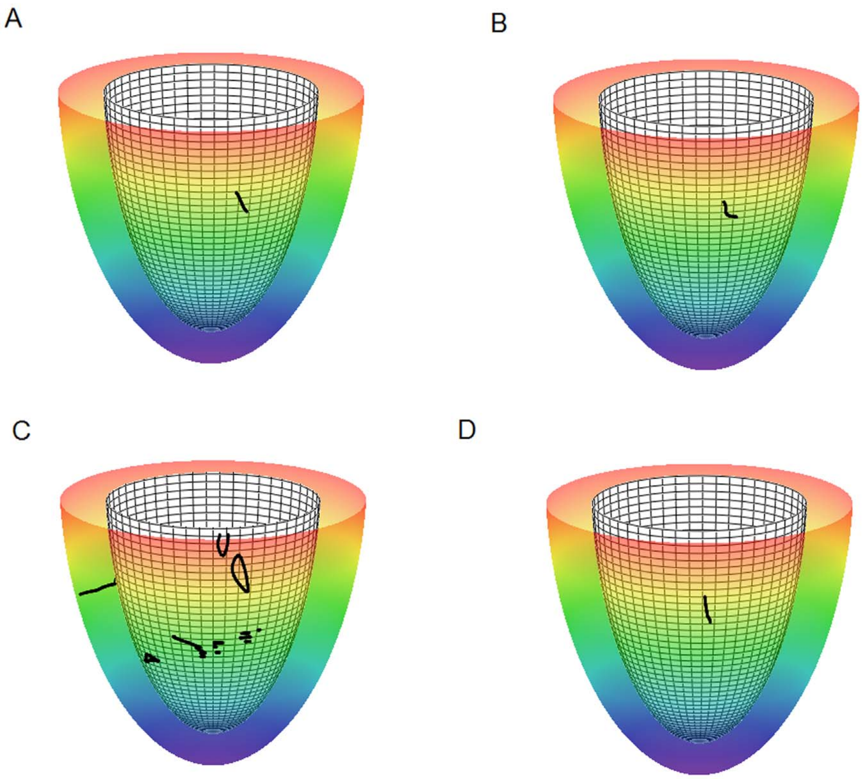

Figure 13. Scroll wave filaments in the LV model. The anisotropy ratio is D_1:D_2 = 1:0.111. Panels A, B, C, D: models 1, 2, 3, 4 (see Table 1), fiber rotation angle in the LV wall increases from panel A to panel D. The epicarduim (semitransparent colored surface; color denotes height from the red base to the purple apex), the endocardium (opaque white meshy surface), and filaments (black lines and dots).
doi:10.1371/journal.pone.0093617.g013

remodeling of the LV geometry might be mimicked in similar mathematical models via the fitting of the geometric parameters values to account for the LV shape of a particular pathology.

The results of our simulations can be verified by direct measurements of wave propagation on the whole heart preparations, such as [53]. Note, however, that another factor important for overall excitation of the heart is the Purkinje conduction system. In order to compare experiments with our simulations, such measurements should be performed after chemical ablation of the Purkinje system [54].

Another potentially interesting application of this approach may be its application in studies on the defibrillation of cardiac tissue, in which case tissue texture and boundary conditions are also of key importance [55]. However, a bi-domain representation of cardiac tissue must be used for defibrillation [55–57], which does not fall under our present scope. Nonetheless, the extension of our approach for such cases is straightforward. The formulae for the diffusion term (7) and for the boundary conditions (9) can be directly used for representation of the bi-domain equations in the special CS. Then, the finite difference problem can be formulated in the same way as in our case and can be solved using any existing method (see [58]).

In this paper, we studied wave propagation due to point stimulation and scroll waves. Other important wave propagation regimes include various types of scroll waves and turbulent patterns [19,59–61]. It was shown that heterogeneity [62,63] and anisotropy [31,64,65] of the tissue are significant factors determining the dynamics of these sources. The effect of heterogeneity on the dynamics of spiral waves was also studied in a series of papers by Shajahan, Sinha and co-authors [30,41,55,67]. In particular, in [66] they showed that some changes in the position, size, and shape of a conduction inhomogeneity can transform a single rotating spiral to spiral breakup or vice versa. Since our model provides tools for changing anisotropic properties and allows one to add heterogeneity, these effects can also be studied using our approach.

Appendices

A Construction of spiral surfaces

We model myocardial sheets as surfaces filling the LV. The filling was obtained by rotation of one surface around the vertical LV symmetry axis. We call these surfaces "spiral" (see Fig. 2).

We introduce the special CS (γ, ψ, ϕ), which is linked with the cylindrical CS (see (1) and (2)). In this CS, the equation of the spiral surface is

$$\phi(\gamma,\psi) = \phi_0 + \phi_{\max}\gamma, \tag{A.1}$$

where different values of ϕ_0 allow us to obtain different spiral surfaces and $\phi_{\max}$ is a constant of the model.

The parametrical equation of a spiral surface in cylindrical CS is

$$\begin{aligned} \rho(\gamma,\psi) &= (r_b + (1-\gamma)l)(\epsilon\cos\psi + (1-\epsilon)(1-\sin\psi)), \\ \varphi(\gamma,\psi) &= \varphi_0 + \varphi_{\max}\gamma, \\ z(\gamma,\psi) &= (z_b + (1-\gamma)h)(1-\sin\psi) + (1-\gamma)h. \end{aligned}$$

B The myofibers' equations

The equations are (see Fig. 3):

$$\rho(\Phi) = \frac{Y}{\sin\Phi}\cdot\left(r_b - \frac{l}{\pi}\Phi\right),$$
$$\phi(\Phi) = \phi_{\max}\Phi/\pi,$$
$$z(\Phi) = z_{ss}(\rho(\Phi),\phi(\Phi)),$$

where different values of the parameter $Y \in (0, 1)$ correspond to different myofibers,

$$\Phi \in [\arcsin Y, \pi - \arcsin Y],$$

$z_{ss}(\rho,\phi)$ is the explicit equation of a spiral surface in the cylindrical CS.

At each point (γ, ψ, ϕ), $0 \le \psi \le \pi/2$, a fiber orientation vector $\mathbf{v} = (x', y', z')$ is given by the formulae:

$$x' = \frac{d\rho}{d\Phi}\cos\phi - \rho\sin\phi\frac{d\phi}{d\Phi}, \tag{B.1}$$

$$y' = -\left(\frac{d\rho}{d\Phi}\sin\phi + \rho\cos\phi\frac{d\phi}{d\Phi}\right), \tag{B.2}$$

$$z' = \frac{\partial z}{\partial\rho}\cdot\frac{d\rho}{d\Phi} + \frac{\partial z}{\partial\phi}\cdot\frac{d\phi}{d\Phi}, \tag{B.3}$$

where ρ is determined by Eq. (1),

$$\Phi = \pi(1-\gamma),$$

$$\frac{d\rho}{d\Phi} = \epsilon_c\cdot\left((r_b + \gamma l)\cot(\gamma\pi) - \frac{l}{\pi}\right),$$

$$\frac{d\phi}{d\Phi} = \frac{\phi_{\max}}{\pi},$$

$$\mathcal{F}' = \frac{\cos\psi}{\epsilon_s},$$

$$\frac{\partial z}{\partial\rho} = \frac{z_b + (1-\gamma)h}{r_b + (1-\gamma)l}\cdot\mathcal{F}',$$

$$\frac{\partial z}{\partial\phi} = \frac{1}{\phi_{\max}}\cdot\left(h\sin\psi + \frac{\mathcal{F}'\epsilon_c l(z_b + (1-\gamma)h)}{r_b + (1-\gamma)l}\right),$$

where $\phi_{\max}$ is a dimensionless parameter affecting fiber twist.

C Laplacian in implicit curvilinear coordinates

The Laplacian is an important term in the reaction-diffusion equation as it is responsible for the modeling of electrical wave spreading. It can be written as div($\mathbf{D}$ grad f) where f is the transmembrane potential and $\mathbf{D}$ is an anisotropic local diffusion matrix.

Below, we calculate the Laplacian for anisotropic diffusion in the Cartesian and the special CS.

C.1 The Cartesian CS. For an arbitrary diffusion matrix $\mathbf{D} = D^{ij}$

$$\mathrm{div}(\mathbf{D}\ \mathrm{grad}\, f) = \sum_{i,j}\frac{\partial D^{ii}}{\partial x_j}\cdot\frac{\partial f}{\partial x_i} + \sum_{i,j} D^{ij}\frac{\partial^2 f}{\partial x_i\,\partial x_j}. \tag{C.1}$$

In consideration of Eq. (6), one can write:

$$\mathrm{div}(\mathbf{D}\ \mathrm{grad}\, f) = (D_1 - D_2)\cdot\sum_{i,j}\frac{\partial(v_i v_j)}{\partial x_j}\cdot\frac{\partial f}{\partial x_i} + \sum_{i,j} D^{ij}\frac{\partial^2 f}{\partial x_i\,\partial x_j} \tag{C.2}$$

C.2 The curvilinear CS. Here we deduce from Eq. (C.2) the proper form of the Laplacian in the curvilinear coordinates ξ_0, ξ_1, ξ_2. Let us consider v_i and f as functions of ξ_0, ξ_1, ξ_2 We calculate three types of derivatives. First, one has

$$\frac{\partial(v_i v_j)}{\partial x_j} = \frac{\partial v_i}{\partial x_j}\cdot v_j + \frac{\partial v_j}{\partial x_j}\cdot v_i, \tag{C.3}$$

where

$$\frac{\partial v_i}{\partial x_j} = \sum_k \frac{\partial v_i}{\partial \xi_k}\cdot\frac{\partial \xi_k}{\partial x_j}. \tag{C.4}$$

Secondly, we evaluate

$$\frac{\partial f}{\partial x_i} = \sum_j \frac{\partial f}{\partial \xi_j}\cdot\frac{\partial \xi_j}{\partial x_i}; \tag{C.5}$$

and thirdly,

$$\frac{\partial^2 f}{\partial x_i\,\partial x_j} = \sum_k \frac{\partial^2 f}{\partial x_i\,\partial \xi_k}\cdot\frac{\partial \xi_k}{\partial x_j}, \tag{C.6}$$

where

$$\frac{\partial^2 f}{\partial x_i\,\partial \xi_k} = \sum_j \frac{\partial^2 f}{\partial \xi_k\,\partial \xi_j}\cdot\frac{\partial \xi_j}{\partial x_i}. \tag{C.7}$$

The difficulty now lies in the fact that the functions $x_j(\xi_k)$ define the ξ_k only implicitly. To evaluate the necessary derivatives, we need the following matrices:

$$\mathbf{J} = (J_{ij}) = \left(\frac{\partial \xi_i}{\partial x_j}\right), \tag{C.8}$$

$$\mathbf{W}=(W_{ij})=\left(\frac{\partial v_i}{\partial x_j}\right),\quad \mathbf{S}=(S_{ij})=\left(\frac{\partial v_i}{\partial \xi_j}\right), \tag{C.9}$$

$$\mathbf{T}^k=(T^k_{ij})=\left(\frac{\partial^2 \xi_k}{\partial x_i\,\partial x_j}\right),\quad \mathbf{H}^k=(H^k_{ij})=\left(\frac{\partial^2 x_k}{\partial \xi_i\,\partial \xi_j}\right). \tag{C.10}$$

The matrices are linked between themselves with the following relations:

$$\mathbf{W}=\mathbf{SJ}, \tag{C.11}$$

$$T^k_{mp}=-\sum_l J_{kl}(\mathbf{J}^T\mathbf{H}^l\mathbf{J})_{mp}.$$

We substitute (C.3), (C.5), (C.7) to (C.2) and get:

$$\mathrm{div}(\mathbf{D}\ \mathrm{grad}\,f)=\sum_k p_k\cdot\frac{\partial f}{\partial \xi_k}+\sum_{k,l} q_{kl}\cdot\frac{\partial^2 f}{\partial \xi_k\,\partial \xi_l},$$

where

$$p_k=D_2\ \mathrm{tr}\,\mathbf{T}^k+(D_1-D_2)\cdot\left((\mathbf{Jv})_k\cdot\mathrm{tr}(\mathbf{SJ})+(\mathbf{JSJv})_k+\mathbf{v}^{\mathrm{T}}\mathbf{T}^k\mathbf{v}\right), \tag{C.12}$$

q_{kl} are elements of matrix $\mathbf{Q}$:

$$\mathbf{Q}=\mathbf{JDJ}^{\mathrm{T}}. \tag{C.13}$$

D Boundary conditions

Let $\mathbf{n}$ be a normal vector to one of the LV boundary surfaces. For outer domain boundaries, we use a no-flux condition on the current.

D.1 Isotropic case, cylindrical CS. One can write the boundary condition as

$$\frac{\partial f}{\partial n}(\rho,\Phi,z)=0. \tag{D.1}$$

By the definition of directional derivative and since the normal vector to the LV boundary lies in our problem always in the corresponding meridional half-plane,

$$\frac{\partial f}{\partial \rho}n_\rho+\frac{\partial f}{\partial z}n_z=0, \tag{D.2}$$

where n_ρ and n_z are the normal vector components in the meridional half-plane.

D.1.1 The equator. On the equator, $n_\rho=0$, so (D.2) reduces to

$$\frac{\partial f}{\partial z}=0. \tag{D.3}$$

D.1.2 The epicardium. On the epicardium, $n_\rho=(z_b+h)\cos\psi$, $n_z=-(r_b+l)\epsilon_s$, so one can write (D.2) as

$$\frac{\partial f}{\partial \rho}\cdot(z_b+h)\cos\psi-\frac{\partial f}{\partial z}\cdot(r_b+l)\epsilon_s=0. \tag{D.4}$$

Everywhere on the epicardium, except the apex,

$$\frac{\partial f}{\partial \rho}=\frac{(r_b+l)\cdot\epsilon_s}{(z_b+h)\cdot\cos\psi}\cdot\frac{\partial f}{\partial z}. \tag{D.5}$$

On the apex, $\cos\psi=0$, $\epsilon_s=\epsilon>0$, so the boundary condition looks like $\frac{\partial f}{\partial z}=0$.

D.1.3 The endocardium. On the endocardium, $n_\rho=z_b\cos\psi$, $n_z=-r_b\epsilon_s$, so (D.2) can be written as

$$\frac{\partial f}{\partial \rho}\cdot z_b\cos\psi-\frac{\partial f}{\partial z}\cdot r_b\epsilon_s=0. \tag{D.6}$$

Everywhere on the endocardium, except the apex,

$$\frac{\partial f}{\partial \rho}=\frac{r_b\epsilon_s}{z_b\cos\psi}\cdot\frac{\partial f}{\partial z}. \tag{D.7}$$

On the apex of the endocardium, like the epicardium, the boundary condition is $\frac{\partial f}{\partial z}=0$.

D.2 Isotropic case, special CS. In the special CS, we use $\frac{\partial f}{\partial \gamma}$ and $\frac{\partial f}{\partial \psi}$. Let us take into account that

$$\frac{\partial f}{\partial \rho}=\frac{\partial f}{\partial \gamma}\cdot\frac{\partial \gamma}{\partial \rho}+\frac{\partial f}{\partial \psi}\cdot\frac{\partial \psi}{\partial \rho} \tag{D.8}$$

and

$$\frac{\partial f}{\partial z}=\frac{\partial f}{\partial \gamma}\cdot\frac{\partial \gamma}{\partial z}+\frac{\partial f}{\partial \psi}\cdot\frac{\partial \psi}{\partial z}. \tag{D.9}$$

D.2.1 The equator. Formula (D.3) can be rewritten as

$$\frac{\partial f}{\partial \psi}=\frac{(r_b+(1-\gamma)l)(1-\epsilon)}{l}\cdot\frac{\partial f}{\partial \gamma}. \tag{D.10}$$

D.2.2 The epicardium. Formula (D.4) becomes

$$\frac{\partial f}{\partial \gamma}=\frac{l(r_b+l)\epsilon_c\epsilon_s-(z_b+h)h\sin\psi\cos\psi}{(z_b+h)^2\cos^2\psi+(r_b+l)^2\epsilon_s^2}\cdot\frac{\partial f}{\partial \psi}. \tag{D.11}$$

D.2.3 The endocardium. Formula (D.6) yields

$$\frac{\partial f}{\partial \gamma}=\frac{lr_b\epsilon_c\epsilon_s-z_bh\sin\psi\cos\psi}{z_b^2\cos^2\psi+r_b^2s^2}\cdot\frac{\partial f}{\partial \psi}. \tag{D.12}$$

Note that if $\epsilon > 0$, the two last formulae cannot have zero values in the denominators and can be directly applied to the LV apex points.

D.3 Anisotropic case, special CS. The boundary condition is

$$\mathbf{nD}\ \mathrm{grad}\, f = 0, \tag{D.13}$$

with $\mathbf{n}$, the normal to the LV surface.

D.3.1 The epi- and endocardium. Let us write (D.13) in detail:

$$\sum_{i,j} n_i D^{ij} \frac{\partial f}{\partial x_j} = 0;$$

$$\sum_{i,j} \left(n_i D^{ij} \cdot \sum_k \frac{\partial f}{\partial \xi_k} \cdot \frac{\partial \xi_k}{\partial x_j} \right) = 0;$$

$$\sum_k \left(\sum_{i,j} n_i D^{ij} \frac{\partial \xi_k}{\partial x_j} \right) \cdot \frac{\partial f}{\partial \xi_k} = 0;$$

$$\mathbf{n}^{\mathrm{T}}\mathbf{DJ}^{\gamma} \frac{\partial f}{\partial \gamma} + \mathbf{n}^{\mathrm{T}}\mathbf{DJ}^{\psi} \frac{\partial f}{\partial \psi} + \mathbf{n}^{\mathrm{T}}\mathbf{DJ}^{\phi} \frac{\partial f}{\partial \phi} = 0. \tag{D.14}$$

Here, the $\mathbf{J}^{\gamma,\psi,\phi}$ are columns of derivatives of these special variables by Cartesian coordinates (see (C.8)):

$$\mathbf{J}^{\gamma} = \begin{pmatrix} \partial\gamma/\partial x \\ \partial\gamma/\partial y \\ \partial\gamma/\partial z \end{pmatrix},$$

and so on.

D.3.2 The equator. Let us write (D.13) detailed as the following:

$$n_x (D\ \mathrm{grad}\, f)_x + n_y (D\ \mathrm{grad}\, f)_y + n_z (D\ \mathrm{grad}\, f)_z = 0. \tag{D.15}$$

Vector $\mathbf{n}$ is collinear to the Oz axis, so $n_x = n_y = 0$. The equation (D.15) goes over

$$(D\ \mathrm{grad}\, f)_z = 0.$$

Writing down the matrix product:

$$\sum_j D^{2j} \frac{\partial f}{\partial x_j} = 0,$$

we substitute (C.5) to this equation:

$$\sum_k \left(\sum_j D^{2j} \frac{\partial \xi_k}{\partial x_j} \right) \frac{\partial f}{\partial \xi_k} = 0.$$

Let us express $\frac{\partial f}{\partial \psi}$:

$$\frac{\partial f}{\partial \psi} = - \frac{\left(D^{20}\gamma_x + D^{21}\gamma_y + D^{22}\gamma_z\right) \frac{\partial f}{\partial \gamma} + \left(D^{20}\phi_x + D^{21}\phi_y + D^{22}\phi_z\right) \frac{\partial f}{\partial \phi}}{D^{20}\psi_x + D^{21}\psi_y + D^{22}\psi_z}.$$

We can calculate the derivatives of γ, ϕ, ψ by x, y, z:

$$\gamma_x = \gamma_\rho \rho_x = -\frac{\cos\phi}{l}; \qquad \gamma_y = \gamma_\rho \rho_y = -\frac{\sin\phi}{l}; \qquad \gamma_z = \frac{r_m(1-\epsilon)}{z_m l};$$

$$\phi_x = -\frac{\sin\phi}{\rho}; \qquad \phi_y = \frac{\cos\phi}{\rho}; \qquad \phi_z = 0;$$

$$\psi_x = \psi_y = 0; \qquad \psi_z = -1/z_m;$$

here, $r_m = r_b + (1-\gamma)l$, $z_m = z_b + (1-\gamma)h$.

So

$$\frac{\partial f}{\partial \psi} = - \frac{\left(-\left(D^{20}x + D^{21}y\right)z_m + D^{22}\rho^2(1-)\right)\frac{\partial f}{\partial \gamma} + z_m l \cdot \left(-D^{20}\sin\varphi + D^{21}\cos\varphi\right)\frac{\partial f}{\partial \varphi}}{D^{22} l \rho}. \tag{D.16}$$

Acknowledgments

The authors are grateful to Arne Defauw for helpful discussions on scroll wave initiation in the ionic model and to Prof. Vitaly I. Berdyshev for useful discussions of the results. Our work was performed using "Uran" supercomputer of IMM UB RAS.

Author Contributions

Conceived and designed the experiments: SP HD LK OS VM AP. Performed the experiments: SP. Analyzed the data: SP HD LK OS VM AP. Wrote the paper: SP HD LK OS AP. Took part in analytical calculations used in this MS: SP HD.

References

1. Noble D (1962) A modification of the hodgkin–huxley equation applicable to purkinje fiber action and pacemaker potential. J Physiol 160: 317–352.
2. Myerburg R, Kessler K, Interian J (1990) Cardiac Electrophysiology: From Cell to Bedside, Philadelphia: Saunders, chapter Clinical and experimental pathophysiology of sudden cardiac death. 1st edition, pp. 666–678.
3. Panfilov A, Holden A, editors (1997) Computational Biology of the Heart. Wiley, Chichester.
4. Panfilov A, Keener J (1995) Re-entry in an anatomical model of the heart. Chaos, Solitons and Fractals 5: 681–689.
5. Panfilov A (1999) Three-dimensional organization of electrical turbulence in the heart. Phys Rev E 59: R6251–R6254.
6. ten Tusscher K, Hren R, Panfilov A (2007) Organization of ventricular fibrillation in the human heart. Circ Res 100: 87–101.
7. Rodriguez B, Li L, Eason J, Efimov I, Trayanova N (2005) Differences between left and right ventricular chamber geometry affect cardiac vulnerability to electric shocks. Circ Res 97: 168–175.
8. Keldermann R, Nash M, Gelderblom H, Wang V, Panfilov A (2010) Electromechanical wavebreak in a model of the human left ventricle. Am J Physiol 299: H134–H143.

9. Jie X, Gurev V, Trayanova N (2010) Mechanisms of mechanically induced spontaneous arrhythmias in acute regional ischemia. CircRes 106: 185–192.
10. Noble D (2002) Systems biology and the heart. Science 295: 1678–1682.
11. ten Tusscher K, Noble D, Noble P, Panfilov A (2004) A model for human ventricular tissue. Am J Physiol Heart Circ Physiol 286: H1573–H1589.
12. ten Tusscher K, Panfilov A (2006) Alternans and spiral breakup in a human ventricular tissue model. Am J Physiol Heart Circ Physiol 291: H1088–1100.
13. Grandi E, Pasqualini F, Bers D (2010) A novel computational model of the human ventricular action potential and ca transient. Journal of Molecular and Cellular Cardiology 48: 112–121.
14. O'Hara T, Virag L, Varro A, Rudy Y (2011) Simulation of the undiseased human cardiac ventricular action potential: Model formulation and experimental validation. PLoS Comput Biol 7: e1002061.
15. Sulman T, Katsnelson L, Solovyova O, Markhasin V (2008) Mathematical modeling of mechanically modulated rhythm disturbances in homogeneous and heterogeneous myocardium with attenuated activity of na+ - k+ pump. Bull Math Biol 70: 910–949.
16. Niederer S, Hunter P, Smith N (2006) A quantitative analysis of cardiac myocyte relaxation: a simulation study. Biophys J 90: 1697–1722.
17. Keurs HT, Shinozaki T, Zhang Y, Zhang M, Wakayama Y, et al. (2008) Sarcomere mechanics in uniform and non-uniform cardiac muscle: a link between pump function and arrhythmias. Prog Biophys Mol Biol 97: 312–331.
18. Rice J, de Tombe P (2004) Approaches to modeling crossbridges and calciumdependent activation in cardiac muscle. Prog Biophys Mol Biol 85: 179–195.
19. Panfilov A, Holden A (1993) Computer simulation of re-entry sources in myocardium in two and three dimensions. Journal of Theoretical Biology 161: 271–85.
20. Aliev R, Panfilov A (1996) A simple two-variable model of cardiac excitation. Chaos, Solitons and Fractals 7: 293–301.
21. Hoogendoorn C, Duchateau N, Sanchez-Quintana D, Whitmarsh T, Sukno F, et al. (2013) A high-resolution atlas and statistical model of the human heart from multislice ct. Medical Imaging, IEEE Transactions on 32: 28–44.
22. Reiser M, Semmler W, Hricak H, editors (2008) Magnetic Resonance Tomography. Springer.
23. Gilbert S, Sands G, LeGrice I, Smaill B, Bernus O, et al. (2012) A framework for myoarchitecture analysis of high resolution cardiac mri and comparison with diffusion tensor mri. In: Engineering in Medicine and Biology Society (EMBC), 2012 Annual International Conference of the IEEE. pp. 4063–4066.
24. Frindel C, Robini M, Rapacchi S, Stephant E, Zhu YM, et al. (2007) Towards in vivo diffusion tensor mri on human heart using edge-preserving regularization. In: Engineering in Medicine and Biology Society, 2007. EMBS 2007. 29th Annual International Conference of the IEEE. pp. 6007–6010.
25. Nielsen P, LeGrice I, Smaill B, Hunter P (1991) A mathematical model of the geometry and fibrous structure of the heart. Am J Physiol 260: H1365–H1378.
26. Trew M, Caldwell B, Sands G, LeGrice I, Smaill B (2011) Three-dimensional cardiac tissue image registration for analysis of in vivo electrical mapping. Ann Biomed Eng 39: 235–248.
27. Pravdin S, Berdyshev V, Panfilov A, Katsnelson L, Solovyova O, et al. (2013) Mathematical model of the anatomy and fibre orientation field of the left ventricle of the heart. Biomedical Engineering Online 54.
28. Pettigrew J (1860) On the arrangement of the muscular fibers of the ventricular portion of the heart of the mammal. Proc Roy Soc, London 10: 433–440.
29. Streeter D (1979) Handbook of physiology. Sec. 2. Vol. I. The Heart, Bethesda, Maryland: Am. Physiol. Soc, chapter Gross morphology and fiber geometry of the heart. pp. 61–112.
30. Sridhar S, Sinha S, Panfilov A (2010) Anomalous drift of spiral waves in heterogeneous excitable media. Phys Rev E 82: 051908.
31. Verschelde H, Dierckx H, Bernus O (2007) Covariant stringlike dynamics of scroll wave filaments in anisotropic cardiac tissue. Physical Review Letters 99: 168104-1–168104-4.
32. Young R, Panfilov A (2010) Anisotropy of wave propagation in the heart can be modeled by a riemannian electrophysiological metric. Proc Natl Acad Sci USA 107: 14964–67.
33. Fenton F, Karma A (1998) Vortex dynamics in three-dimensional continuous myocardium with fiber rotation: Filament instability and fibrillation. Chaos: An Interdisciplinary Journal of Nonlinear Science 8: 20–47.
34. Kawel N, Turkbey EB, Carr JJ, Eng J, Gomes AS, et al. (2012) Normal left ventricular myocardial thickness for middle-aged and older subjects with steady-state free precession cardiac magnetic resonance: The multi-ethnic study of atherosclerosis. Circulation: Cardiovascular Imaging 5: 500–508.
35. Andre F, Lehrke S, Katus H, Steen H (2012) Reference values for the left ventricular wall thickness in cardiac mri in a modified aha 17-segment model. Journal of Cardiovascular Magnetic Resonance 14 (Suppl. 1): P223.
36. Kaul S, Wismer G, Brady T, Johnson D, Weyman A, et al. (1986) Measurement of normal left heart dimensions using optimally oriented mr images. American Journal of Roentgenology 146: 75–79.
37. Caldwell BJ, Trew ML, Sands GB, Hooks DA, LeGrice IJ, et al. (2009) Three distinct directions of intramural activation reveal nonuniform side-to-side electrical coupling of ventricular myocytes. Circulation: Arrhythmia and Electrophysiology 2: 433–440.
38. Saffitz JE, Kanter HL, Green KG, Tolley TK, Beyer EC (1994) Tissue-specific determinants of anisotropic conduction velocity in canine atrial and ventricular myocardium. Circulation Research 74: 1065–1070.
39. Kleber AG, Rudy Y (2004) Basic mechanisms of cardiac impulse propagation and associated arrhythmias. Physiol Rev 84: 431–488.
40. Cross MC, Hohenberg PC (1993) Pattern formation outside of equilibrium. Rev Mod Phys 65: 851–1112.
41. Nayak AR, Shajahan TK, Panfilov AV, Pandit R (2013) Spiral-wave dynamics in a mathematical model of human ventricular tissue with myocytes and fibroblasts. Physiol Rev 8: e72950.
42. Berenfeld O, Wellner M, Jalife J, Pertsov A (2001) Shaping of a scroll wave filament by cardiac fibers. Phys Rev E Stat Nonlin Soft Matter Phys 63: 061901.
43. Wellner M, Berenfeld O, Jalife J, Pertsov AM (2002) Minimal principle for rotor filaments. Proceedings of the National Academy of Sciences 99: 8015–8018.
44. Davydov V, Morozov V, Davydov N, Yamaguchi T (2004) Propagation of autowaves in excitable media with chiral anisotropy. Physics Letters A 325: 334–339.
45. Carapella V, Bordas R, Pathmanathan P, Schneider J, Kohl P, et al. (2013) Functional Imaging and Modelling of the Heart, Springer, chapter Effect of fibre orientation optimisation in an electromechanical model of left ventricular contraction in rat. pp. 46–53.
46. Qu Z, Kil J, Xie F, Garfinkel A, Weiss JN (2000) Scroll wave dynamics in a threedimensional cardiac tissue model: Roles of restitution, thickness, and fiber rotation. Biophysical Journal 78: 2761–2775.
47. Misner C, Thorne K, Wheeler J (1973) Gravitation. W.H. Freeman and Co.
48. Zykov V, Müller S (1996) Spiral waves on circular and spherical domains of excitable medium. Phys D 97: 322–332.
49. Dierckx H, Brisard E, Verschelde H, Panfilov AV (2013) Drift laws for spiral waves on curved anisotropic surfaces. Phys Rev E 88: 012908.
50. Trayanova NA, Boyle PM (2013) Advances in modeling ventricular arrhythmias: from mechanisms to the clinic. Wiley Interdisciplinary Reviews: Systems Biology and Medicine.
51. Samie FH, Jalife J (2001) Mechanisms underlying ventricular tachycardia and its transition to ventricular fibrillation in the structurally normal heart. Cardiovascular Research 50: 242–250.
52. Ramrakha P, Hill J, editors (2012) Oxford Handbook of Cardiology. Oxford University Press, 2nd edition.
53. Taccardi B, Macchi E, Lux RL, Ershler PR, Spaggiari S, et al. (1994) Effect of myocardial fiber direction on epicardial potentials. Circulation 90: 3076–90.
54. Dosdall D, Tabereaux P, Kim J, Walcott G, Rogers J, et al. (2008) Chemical ablation of the purkinje system causes early termination and activation rate slowing of long-duration ventricular fibrillation in dogs. Am J Physiol Heart Circ Physiol 295: H883–9.
55. Keener J, Panfilov A (1996) A biophysical model for defibrillation of cardiac tissue. Biophys J 71: 1335–45.
56. Henriquez C (1993) Simulating the electrical behavior of cardiac tissue using the bidomain model. Crit Rev Biomed Eng 21: 1–77.
57. Clayton R, Bernus O, Cherry E, Dierckx H, Fenton F, et al. (2011) Models of cardiac tissue electrophysiology: Progress, challenges and open questions. Progress in Biophysics and Molecular Biology 104: 22–48.
58. Vigmond E, dos Santos RW, Prassl A, Deo M, Plank G (2008) Solvers for the cardiac bidomain equations. Progress in Biophysics and Molecular Biology 96: 3–18.
59. Panfilov A (1999) Three-dimensional organization of electrical turbulence in the heart. Physical Review E 59: R6251–R6254.
60. Panfilov A, Holden A (1990) Self-generation of turbulent vortices in a 2-dimensional model of cardiac tissue. Physics Letters A 151: 23–26.
61. Panfilov A, Winfree A (1985) Dynamical simulations of twisted scroll rings in threedimensional excitable media. Physica D 17: 323–30.
62. Rudenko A, Panfilov A (1983) Drift and interaction of vortices in two-dimensional heterogeneous active medium. Studia Biophysica 98: 183–88.
63. Panfilov A, Vasiev B (1991) Vortex initiation in a heterogeneous excitable medium. Physica D: Nonlinear Phenomena 49: 107–13.
64. Panfilov A, Keener J (1993) Generation of re-entry in anisotropic myocardium. J Cardiovasc Electrophysiol 4: 412–421.
65. Panfilov A, Holden A (1990) Vortices in a system of two coupled excitable fibers. Physics Letters A 147: 463–466.
66. Shajahan T, Sinha S, Pandit R (2007) Spiral-wave dynamics depend sensitively on inhomogeneities in mathematical models of ventricular tissue. Phys Rev E Stat Nonlin Soft Matter Phys 75: 011929.
67. Sinha S, Stein KM, Christini DJ (2002) Critical role of inhomogeneities in pacing termination of cardiac reentry. Chaos: An Interdisciplinary Journal of Nonlinear Science 12: 893–902.
